# Citizen Characteristics and Their Participation in Food Safety Social Co-governance: Public Health Implications

**DOI:** 10.3389/fpubh.2021.772117

**Published:** 2021-11-24

**Authors:** Xiujuan Chen, Ke Qin, Linhai Wu

**Affiliations:** ^1^School of Business, Jiangnan University, Wuxi, China; ^2^Institute for Food Safety Risk Management, Jiangnan University, Wuxi, China

**Keywords:** citizen characteristics, psychological capital, food safety social co-governance, analysis of variance, *post-hoc* multiple comparisons

## Abstract

**Objective:** Given that positive psychological capital motivates citizens to actively participate in social affairs, this study aims to provide insight into food safety risk management in China by empirically determining which individual characteristics are associated with positive psychological capital for actively participating in social co-governance.

**Methods:** A questionnaire-based survey was undertaken between December 5 and 10, 2020. The study participants were residents of Wuxi in China over the age of 18 years. A validated and pretested questionnaires was used to elicit responses with the participants. Student's *t*-test and one-way analysis of variance were performed to determine which individual characteristics are significantly correlated with the psychological capital of citizens who participate in co-governance. *Post-hoc* multiple comparisons were performed for each individual characteristic with a significant correlation to determine which categories of these characteristics yielded the significant differences. Study data were analyzed using IBM SPSS Statistics 24.0.

**Results:** A total of 752 completed responses were received. Most respondents were females (52.39%), aged 26–45 (66.09%), married (70.48%), company employees (44.28%), and in good health (89.76%). Most had a household size of 3 (55.98%), a bachelor's degree (40.96%), a personal annual income of more than 100,000 yuan (26.46%), and no children aged under 18 (50.27%) or pregnant women (93.22%) in their households. Data analysis indicated that education, income, and health status significantly associate with the psychological capital of citizens to participate in co-governance. Citizens with high education, high income, and good health status have higher psychological capital to participate in co-governance.

**Conclusion:** The present study suggested citizens are likely to actively participate in food safety social co-governance only when they have at least one of the following three characteristics: (1) higher than average income in their city of residence; (2) a bachelor's degree or higher education; or (3) good health. Therefore, motivating citizens to participate in co-governance is a long-term process in China. The fundamental strategy is to increase the income of citizens, especially among low-income groups, promote education to improve the food safety literacy of the public, and improve sanitation and public health.

## Introduction

Food safety is a major global public issue (1). Numerous studies have shown that it is difficult to avoid government or market failure, either alone or combined, by relying solely on the government or market, or on both government and market together to manage food safety risks. The traditional governance model has been unable to effectively meet the consumption needs of the society (2, 3). Ensuring food safety is the common responsibility of all stakeholders (4). In fact, the phenomenon of social co-governance first emerged in the 1960s and 1970s and since developed as a new governance model in Western countries. It has now become the most basic model for managing food safety risks in Western countries (5, 6). Although China has different national conditions from Western countries, the Chinese government has begun to reform the country's governance model over the last two decades, especially since 2012 (7), and the revised Food Safety Law has established social co-governance as the basic principle for managing food safety risks. At present, the academic community generally agrees that food safety social co-governance means that stakeholders, including the government, enterprises (market), social organizations, and citizens, jointly formulate or participate in the formulation of laws, regulations, and rules, coordinate and cooperate with each other, and fulfill their respective responsibilities in accordance with the law to jointly ensure food safety with low governance costs in an open, transparent, and flexible manner (8–10).

In a general sense, a citizen is a person who has citizenship and thus has certain rights as well as obligations in accordance with the laws of that country. According to Baidu Encyclopedia, it represents the concept of an individual, whereas the public is a collection of individual citizens and also includes legal persons and other organizations. Richard (11) argued that citizens are the best judges of their own behavior. Citizens capable of independent behavior are not simply food consumers. They should not only bear the responsibility of self-protection (12), but can also act as the best regulators of food safety. Following the inclusion of food safety in China's national security system in 2011, the food safety co-governance system has been gradually improved, and a consensus has gradually emerged regarding the role of social co-governance; the concept of “citizen,” with its connotation of responsibilities as well as rights, reflects the internal logic of this modernization of China's national governance system and governance capabilities. As such, they are an important force in social co-governance, playing an irreplaceable role (11, 13). For example, citizens can provide tip-offs to the media and regulatory agencies, report complaints and violations, and use the media to urge the government to strengthen food safety regulation. In this way, they play a unique role in improving food safety and become an important force in social co-governance (14, 15).

Studies in Western countries have shown that individual psychological capital (PsyCap) can ignite positive emotions in citizens and motivate them to actively participate in public affairs (10, 16), thus constituting the micro-psychological foundation of food safety social co-governance (10). Unlike Western countries, food safety incidents still occur frequently in China at its current stage of development, and food safety remains one of the top concerns among Chinese citizens (17). However, at present, most Chinese citizens merely play the role of passive consumer (18), and do not actively participate in co-governance (19). Starting from the practice in Western countries and the concept of PsyCap, how can the positive emotions of Chinese citizens to participate in co-governance be ignited and their PsyCap be improved? What are the individual characteristics of citizens who have high PsyCap and will actively participate in social co-governance? These questions have not been adequately answered in the literature. To this end, this study aims to offer an exploratory introduction of the concept PsyCap and its four dimensions, establish an analytical framework for the relationship between the characteristics and PsyCap of citizens who participate in co-governance, and examine the differences in the PsyCap of citizens with different individual characteristics based on a micro-level survey. The results of this study may provide a scientific basis for understanding how to motivate citizens to actively participate in social co-governance, and thus promote the construction of a new mechanism for food safety social co-governance in China.

## Literature Review: Meaning of PsyCap and Relationship Between PsyCap and Citizen Characteristics

The concept of PsyCap first appeared in the economics and sociology literature. However, it was long believed that PsyCap was difficult to measure due to its instability, variability, and tendency to undergo dynamic changes (20). Only after Goldsmith et al. (21) found a correlation between an individual's PsyCap and income did PsyCap begin to be measurable, developable, and manageable. However, Goldsmith et al. (21) did not clearly define the concept of PsyCap. To address the situation whereby the field of psychology paid too much attention to human weakness and negative psychology, Seligman (22) proposed the concept of positive psychology, trying to direct psychology research to harness the power of progress and social virtues. This groundbreaking idea triggered intense research on PsyCap and its relationship with positive behavior. Avolio et al. (23) believed that PsyCap is a positive psychological state that affects individual emotions and improves their behavior. Luthans et al. (24) further proposed the concept of positive PsyCap (referred to simply as PsyCapfor short hereafter) based on the inclusion criteria of positive organizational behavior (i.e., being positive, measurable, open to development, and performance-influencing, and having a theoretical rationale) and defined it as a positive psychological state shown by an individual in the process of growth and development. It is the core psychological element that transcends human and social capital, and is a psychological resource that promotes personal growth.

Although the concept of PsyCap has not yet been adequately studied, it can be understood from the following three perspectives based on existing research. The first perspective is trait theory, which suggests that PsyCap is an inherent trait of individuals. Hosen et al. (25) believed that PsyCap is a basic psychological quality that is durable, relatively stable, and acquired through long-term investment, such as learning. Casey and Grzywacz (26) equated PsyCap with personality, which is a result of both nature and nurture. The second perspective is state theory, which posits that PsyCap is a positive psychological state that affects individual behavior, and is a complex culmination of various individual psychological factors (27). Goldsmith (21) pointed out that PsyCap includes self-perception, attitudes toward work, ethical orientation, and general outlook on life. Tettegah (28) understood PsyCap as a complex phenomenon formed by the interaction of self-perception, attitudes toward work, ethical orientation, beliefs about life, values, and consciousness;PsyCap is seen as having the ability to change behavior by influencing an individual's psychological state. The third perspective is the synthesis theory, which suggests that PsyCap is a state-like resource and a psychological quality with characteristics of both trait and state that can be effectively developed in a specific manner. At present, synthesis theory has predominated in terms of understanding PsyCap (29).

Since the start of the twenty-first century, the study of PsyCap has been extended to the field of human resource management. Ke et al. (30) found that male employees generally had higher PsyCap than female employees, and that age and PsyCap were generally positively correlated. Fang (31) pointed out that adolescents' PsyCap increased with age. Sui et al. (32) found that individual characteristics, such as education and years of working, were correlated with PsyCap and job performance. Babalola (33) suggested that individuals with higher education were more likely to have higher levels of self-confidence and optimism. Cole et al. (34) found that individuals with higher economic income and social status had higher PsyCap. Ke et al. (35) reported that age, gender, education, and years of working all had a significant impact on the PsyCap of Chinese employees.

However, other studies have found no significant correlation between individual characteristics and PsyCap. For example, Avey (36) used 1,264 engineers from American Airlines and 524 technicians from China Telecom as comparative samples. Among the US employees, although age was shown to be correlated with PsyCap, no distinct differences in PsyCap caused by age, gender, or employment duration were observed. Among the Chinese employees, age, gender, or employment duration did not significantly affect PsyCap. Similarly, Luthans et al. (37) found no significant correlation of PsyCap with gender, age, or education. Likewise, Mathe-Soulek et al. (38) found no significant correlation between individual characteristics, such as gender and age, and PsyCap in a study of employee performance in the restaurant industry.

The above literature review discussed the nature of PsyCap in terms of trait, state, and synthesis. However, PsyCap has a very rich meaning. In terms of meaning, it is agreed that PsyCap is both a state and a trait. It is neither purely a state, like emotions, i.e., something transient, variable, and unstable, nor purely a trait, like personality and physical characteristics, which are difficult to change. Instead, it is state-like and can be developed through interventions (9–40). Moreover, the literature on PsyCap in the field of human resource management shows the existence of many relationships between PsyCap and individual characteristics of citizens; these relationships are not fixed, but vary with the public affairs and external scenarios involved. However, no answer has been provided to the question of how to measure the relationship between individual characteristics and PsyCap of citizens who participate in food safety social co-governance. Therefore, it may be worth exploring the topic to establish a framework for analyzing the relationship between characteristics and PsyCap of citizens who participate in co-governance based on their PsyCap composition.

## A Framework for Analyzing the Relationship Between Characteristics and PsyCap of Citizens Participating in Co-governance

Luthans et al. (41) suggested that self-efficacy, resilience, optimism, and hope are the four most basic core elements that constitute measurable state-like PsyCap and significantly affect individual attitudes and behaviors. These factors may provide insight into developing a framework for analyzing the relationship between characteristics and PsyCap of citizens who participate in co-governance.

Stajkovic et al. (42) defined self-efficacy as “an individual's convictions (or confidence) about his or her abilities to mobilize the motivation, cognitive resources, and courses of action needed to successfully execute a specific task within a given context.” According to Parker and Sharon (43), self-efficacy refers to an individual's confidence about their abilities to face challenges, do their job, and strive to succeed. In terms of food safety, as the main topic of the present study, a survey by Ovca et al. (44) showed that most students in Sloveniawere confident in handling food safely. Haapala and Probart (45) suggested that middle school students in central Pennsylvania had high self-efficacy in food handling and food safety issues. Moreover, females had significantly higher self-efficacy than males, and this gap increased with age. Similarly, Richards and Beavers (46) found that adolescents in six southeastern US states were confident in their ability to influence food safety and exhibited strong self-efficacy. Schafer et al. (47) investigated the impact of different individual characteristics, including age, gender, marriage status, household size, employment status, education, and income, on food safety attitudes and behaviors by using them as independent variables. They found that individuals who were female, elderly, or had a large household size had higher self-efficacy and were more likely to actively participate in food safety risk management. Cater et al. (48) suggested pregnant women were highly confident in knowing how to keep foods safe for consumption based on a random survey of 222 pregnant women from Louisiana and surrounding areas in the United States. Gase et al. (49) found that increasing the availability of healthy food helped to increase people's self-efficacy regarding their ability to eat healthy.

Resilience is the ability of individuals, families, or groups to quickly recover from adversity, setbacks, and failures and actively change their mentality (50). Bestor (51) and Rosenberger (52) suggested that due to doubts about imported food, many citizens in Japanoften turn to domestic products as a safer and more reliable substitute for imported food. Khanna et al. (53) and Lutz et al. (54) found that better diet quality, which means higher intake of vegetables and fruits, was associated with higher resilience. Whatnall et al. (55) found that a healthy diet helped improve psychological health and resilience in a survey of Australian college students. That study further found that college students with an unhealthy diet had poorer psychological health than the average person. Ren (56) pointed out that individuals with higher education and income levels were more sensitive to food safety, more cautious about food safety incidents, and able to manage their panic. Wang et al. (57) found a slower recovery of consumer confidence among citizens with higher income. They suggested this may be because the consumer confidence of high-income households is more severely affected by food safety incidents and more difficult to restore due to their original higher trust in food safety. Ren and Han (58) suggested that resilience is related to occupation and that food safety incidents do not significantly change the consumption of that category of food by individuals in food-related occupations. Likewise, Li et al. (59) reported that after the 2008 melamine-tainted milk powder crisis in China, Chinese citizens with more children or lower incomes had higher resilience than those with fewer children or higher incomes and were more confident in domestic infant milk powder.

Optimism is a positive attitude toward current and future success expectations (60). Optimistic individuals have less anxiety and are more inclined to express happy emotions; positive expectations for the future indicate their inner confidence and are the source of optimism (61). Jonge et al. (62) defined individual confidence in food safety as a psychological attitude that food safety is generally controllable and will not harm their health. A series of food safety incidents in China, such as the Shineway clenbuterol crisis, the cadmium-contaminated rice incident in Hunan province, and the Shanghai Fuxi food incident, have affected the confidence of Chinese citizens in future food safety (63). Ren et al. (64) surveyed Chinese consumers of different classes and found that individuals with an older age, higher education, or higher income had a higher food safety knowledge and lower confidence in food safety in China. According to Wang and Gu (65), more than 70% of Chinese citizens believed that Western developed countries had a higher level of food safety. A survey by Cheng et al. (66) found that middle school students in Beijing were pessimistic about food safety in China. Jonge et al. (62) reported that individuals with higher education were more optimistic about food safety, but the elderly were more pessimistic. Jonge et al. (67) also found a significant positive correlation between education and optimism about food safety among Canadian and Dutch consumers.

Hope provides individuals with lasting beliefs, positive expectations, and motivation for sustained efforts to achieve their goals (68). Liu et al. (69) reported that highly-educated female citizens aged 35–54 years expressed the strongest desire for the right to know about genetically modified foods. Zhang and Zhang (70) argued that gender, age, education, income, and household size are important factors that affect individual expectations for future food safety. Nan et al. (71) found that citizens with poor health were more sensitive to food safety risks due to concerns about their own health. Ye (72) suggested that individuals with higher education and income paid more attention to health, were more willing to search for food safety information and learn how to identify safe food, and were more aware of protecting their own rights when experiencing food safety issues. Sternsdorff-Cisterna (73) found that after the end of nuclear radiation crisis in Japan, Japanese women with children were skeptical of the government's commitment to food safety and were more willing to participate in food safety risk management.

To date, few studies have directly investigated the relationship between individual characteristics and PsyCap of citizens willing to participate in co-governance. However, research in other related fields provides a useful reference for thinking about this unexplored issue. Caprara et al. (74) found that the self-efficacy of Italian voters in political participation varied with gender, age, education, and income, and that voters participated actively only when they thought they were capable of handling political affairs. Zani and Barrett (75) reached a similar conclusion, and pointed out that the enthusiasm of citizens for political participation was not only affected by individual psychological factors, but also by the macro-environment. Crocetti et al. (76) found that US youths had higher political self-efficacy and higher levels of involvement in political activities than Italian youths. Zhao et al. (77) included gender, age, marital status, and education as control variables, and found that the PsyCap of college teachers to participate in public affairs was significantly related to age and education. Wang and Jiang (78) reported that female farmers were more willing to participate in the joint management of agricultural product safety risks than male farmers. Xu et al. (79) suggested that the PsyCap of migrant workers was significantly influenced by years of education, health status, and income. Jang (80) found that government employees aged over 40 years had higher PsyCap to participate in public affairs than younger ones. Therefore, there may be a correlation between occupation and the PsyCap to participate in social governance.

Citizens can be considered the basic unit in food safety risk management. In general, citizens have different consumption attitudes and food safety perceptions due to differences in individual characteristics such as gender, age, income, education, and household size (81, 82). Nayga (83) investigated the relationship between individual characteristics and attention to food safety and found that housewives with low education and income were more likely to perceive food safety risks. Baker (84) suggested that women and individuals with children in the household were most likely to avoid food safety risks. Flynn et al. (85) believed that gender was an important factor affecting individual perception of food safety risks. Both Dosman et al. (86) and Finke and Kim (87) confirmed the finding of Flynn et al. (85), and suggested that females were more concerned about food safety than males. Sternsdorff-Cisterna (73) reported that nearly 20% more Japanese females than males thought that nuclear radiation would cause serious harm to food safety, and thus argued that females were more willing to participate in food safety co-governance.

Dosman et al. (86) suggested that age is also a factor affecting food safety attitudes, and that the elderly are more worried about the health threats of pesticide residues in food. Roe et al. (88) pointed out that households with low income and education showed higher risk aversion and paid more attention to food safety. Wu et al. (89) believed that public perception of food safety risks is significantly related to individual characteristics such as age, gender, education, and income. According to Luthans (90), individual characteristics influence the PsyCap of citizens by acting on self-efficacy, resilience, optimism, and hope. Therefore, it can be argued that the differences in the perception and attitudes of citizens with different individual characteristics regarding food safety issues are attributable to differences in individual PsyCap. However, there are few studies that systematically examine the relationship among individual characteristics, PsyCap, and attitudes toward participation in food safety social co-governance.

**Figure 1:**
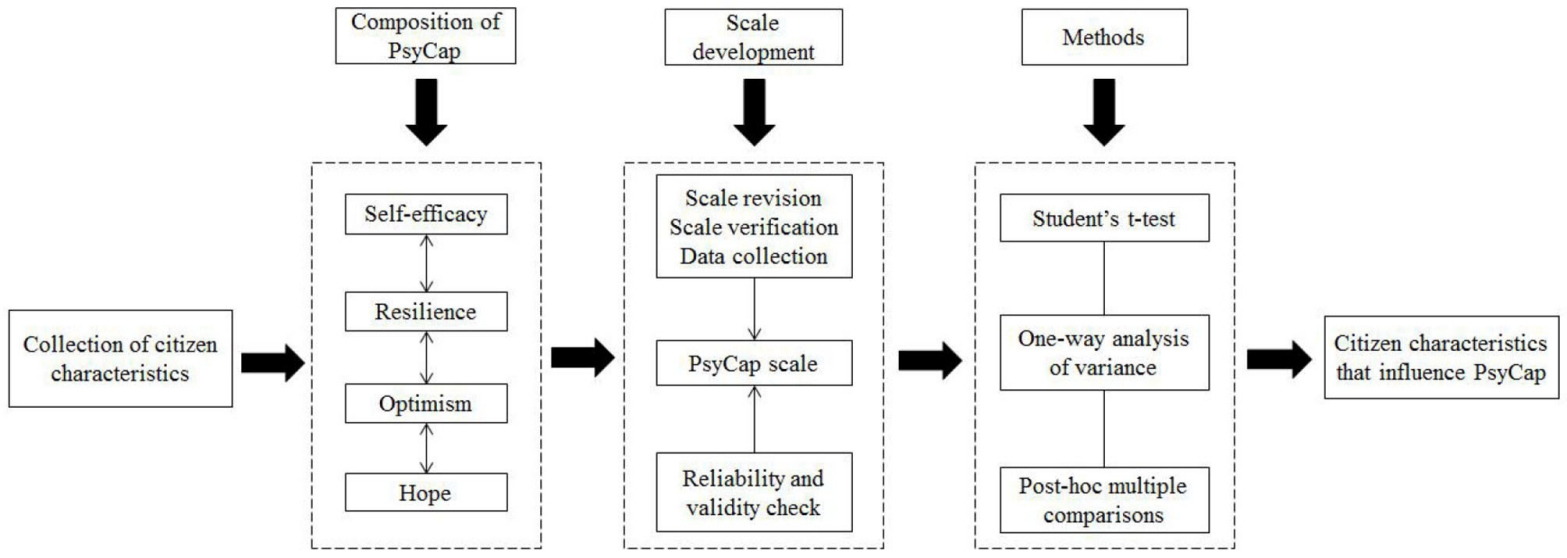
Framework for analyzing the relationship between characteristics and PsyCap of citizens who participate in social co-governance.

In summary, numerous pioneering studies have been conducted on individual characteristics that associate with PsyCap. However, most of these studies only investigated the correlation between one or several individual characteristics and PsyCap. Studies have rarely incorporated age, income, education, marriage, occupation, gender, health status, and household size into a system to construct a collection of characteristics and comprehensively examine the correlation among several individual, family, and social characteristics in the collection and PsyCap. Furthermore, no studies have presented a complete framework for analyzing the relationship between characteristics and PsyCap of citizens. Therefore, drawing on existing literature, this study develops a 16-item scale to measure the PsyCap of citizens who participate in food safety social co-governance based on the four dimensions that are generally believed to constitute PsyCap, namely self-efficacy, resilience, optimism, and hope (90). Moreover, the scale is revised based on a pre-survey and tested for reliability and validity. On this basis, the effects of citizen characteristics on the four dimensions are analyzed using Student's *t*-test, one-way analysis of variance (ANOVA), and *post-hoc* multiple comparisons to examine the relationship between the characteristics and PsyCap of citizens who participate in food safety social co-governance. Based on the meaning and four-dimensional composition of PsyCap, this study develops the original analytical framework as shown in Figure 1 to thoroughly examine the relationship between the characteristics and PsyCap of citizens who participate in food safety social co-governance. This framework clearly describes the process used in this study to explore ways to improve the psychological level of citizens to increase active participation in social co-governance.

## Survey Design, Research Methods, and Sample Statistics and Tests

### Sample Selection

In 2020, Wuxi in Jiangsu Province had the highest per capita GDP among all cities in China. Given the area's relatively balanced economic and social development, citizens in Wuxi show relatively high satisfaction with food safety, which provides a good foundation for this research. Primary data were collected from a field questionnaire survey in the five administrative districts (Liangxi, Xishan, Huishan, Binhu, and Xinwu) in Wuxi. The sample size in each district was proportional to the respective resident population. The survey was carried out among citizens aged over 18 years (herein after referred to as respondents) in farmers' markets and chain supermarkets with a large flow of customers. Investigators were instructed to select the third person coming into view as a respondent to ensure the randomness of sampling as much as possible (91). The questionnaires were completed anonymously by respondents at the survey sites and collected once completed. The entire survey process was completed in the period December 5–10, 2020. In total, 752 valid samples were obtained.

### Questionnaire Design

A questionnaire survey provides authenticity and validity for assessing the psychological characteristics, attitudes, and behaviors of respondents (92, 93). Various classification methods have been proposed for the structure of PsyCap, mainly including two- (94), three- (21, 95), four- (96), and multi-dimensional structures (97). Among them, the four-dimensional classification method of Luthans et al. (24) has been widely accepted. According to Luthans et al. (24), psychological capital is composed of four dimensions, i.e., self-efficacy, resilience, optimism, and hope. Moreover, Luthans et al. (96) developed a questionnaire consisting of 24 items to measure PsyCap based on these four dimensions, which has been widely used in human resource management. Therefore, this study develops a scale for measuring the PsyCap of citizens who participate in food safety social co-governance, which includes four dimensional subscales in Table 1, drawing on the research of Luthans et al. (96) based on the analytical framework shown in Figure 1. Each subscale is composed of four items.

**Table 1:** Scale for PsyCap of citizens in food safety social co-governance.

**Subscale**	**Item**
Self-efficacy	1. You are confident in the safety of the food you buy.
	2. You are confident to solve food safety problems you may encounter.
	3. You cannot obtain accurate food safety information.
	4. You believe that you can contribute to joint solving of food safety problems.
Resilience	5. You learn from any food safety accidents you have experienced, if any, and improve your food safety awareness afterwards.
	6. You seek a solution calmly when encountering food problems.
	7. You take great care to avoid buying defective food products.
	8. You have to a zero-tolerance attitude toward foods with safety risks.
Optimism	9. You believe that citizens can play a role in food safety risk management.
	10. You believe that the quality of the vast majority of food in the market is guaranteed.
	11. You believe that food safety is better now than in the past.
	12. You believe that food safety will be better in the future.
Hope	13. You believe that it is meaningful to participate in food safety social co-governance.
	14. You believe that food safety will be better in the future despite the persistent occurrence of food safety incidents at present.
	15. You will actively learn food safety knowledge to protect your dietary health.
	16. You believe that there will be better solutions to food safety issues in the future.

A 5-point Likert scale was used where 1 = strongly disagree and 5 = strongly agree. The higher the score, the higher the PsyCap, except for Item 3, which has reverse scoring. The characteristic variables and their values are presented in Table 2.

**Table 2:** Independent variables.

**Variable**	**Value**	**Mean**	**Standard deviation**
Gender	Male = 1; female = 2	1.52	0.500
Age	18–25 years = 1; 26–35 years = 2; 36–45 years = 3; 46–55 years = 4; 56 years and older = 5	2.46	1.017
Marital status	Married = 1; unmarried = 2	1.30	0.456
Household size (n)	1 person = 1; 2 persons = 2; 3 persons = 3; 4 persons = 4; 5 persons and more = 5	3.34	0.854
Education	Junior high school or lower = 1; high school = 2; junior college = 3; bachelor's degree = 4, master's degree or higher = 5	3.33	1.107
Personal annual income	36,000 yuan or lower = 1; 36,000–50,000 yuan = 2; 50,000–80,000 yuan = 3; 80,000–100,000 yuan = 4; 100,000 yuan or higher = 5	3.19	1.469
Having children aged under 18 in the household	No = 1; Yes = 2	1.50	0.500
Having a pregnant woman in the household	No = 1; Yes = 2	1.07	0.252
Health status	Good = 1; fair = 2; poor = 3	1.11	0.349
Occupation	Government or public institution employee = 1; company employee = 2; farmer = 3; school students = 4; others = 5	2.74	1.328

### Analysis Methods

Data were processed using SPSS 24.0 software. Mean difference was used to analyze the association between different individual characteristics and the PsyCap of citizens who participate in food safety social co-governance. Student's *t*-test was used for dichotomous categorical variables and ANOVA for multichotomous variables. A significant *F*-value from ANOVA indicates that there are significant differences in the means of the dependent variable between at least two categories of the independent variable. In this case, *post-hoc* multiple comparisons were performed to determine which categories yielded the significant differences (98). *Post-hoc* multiple comparisons are used to compare the means of three or more categories to further determine the differences between each two categories (99). *Post-hoc* comparisons were performed *via* the least significant difference (LSD) test when the homogeneity of variance assumption was met. Otherwise, Tamhane's T2-test was employed.

### Demographics of Respondents

Respondent demographics are presented in Table 3. In terms of gender composition, there were more females than males, which is consistent with the fact that in China, most household food purchasers are female. Most respondents were aged 26–45 (66.09%), married (70.48%), company employees (44.28%), and in good health (89.76%). In addition, most had a household size of 3 (55.98%), a bachelor's degree (40.96%), and a personal annual income of more than 100,000 yuan (26.46%). Finally, most had no children aged under 18 (50.27%) or pregnant woman (93.22%) in their households. It should be noted that the demographics of the sample do not perfectly match the overall demographics of Wuxi due to the higher number of females surveyed than males. However, this does not compromise the representativeness of the survey sample because household food is mostly purchased by one or some family members, who are generally female. Given the sampling method used, i.e., surveying carried out at the point of purchase of food, this sample distribution therefore reflects food purchase behavior in the study area. In fact, the demographics of the study's sample are consistent with those of Wu et al. (100) and Wu et al. (101), which are previous studies conducted in the same survey area as this study. However, readers should assess the results of this study based on consideration of data representativeness.

**Table 3:** Demographics of respondents.

**Demographic**	**Category index**	**Sample size (*n*)**	**Proportion (%)**
Gender	Male	358	47.61
	Female	394	52.39
Age	18–25 years	143	19.02
	26–35 years	252	33.51
	36–45 years	245	32.58
	46–55 years	92	12.23
	56 years or older	20	2.66
Marital status	Married	530	70.48
	Unmarried	222	29.52
Household size (*n*)	1	13	1.73
	2	63	8.38
	3	421	55.98
	4	167	22.21
	5 or more	88	11.70
Education	Junior high school or lower	60	7.98
	High school (including vocational high school)	111	14.76
	Junior college (including higher vocational colleges)	187	24.87
	Bachelor's degree	308	40.96
	Master's degree or higher	86	11.44
Personal annual income	<36,000 yuan	144	19.15
	36,000–50,000 yuan	120	15.96
	50,000–80,000 yuan	133	17.69
	80,000–100,000 yuan	156	20.74
	More than 100,000 yuan	199	26.46
Having children aged under 18 in the household	No	378	50.27
	Yes	374	49.73
Having a pregnant woman in the household	No	701	93.22
	Yes	51	6.78
Health status	Poor	8	1.06
	Fair	69	9.18
	Good	675	89.76
Occupation	Government or public institution employee	119	15.82
	Company employee	333	44.28
	Farmer	26	3.46
	Student	98	13.03
	Others	176	23.40

### Sample Tests

#### Reliability Test

Scale reliability indicates the consistency of results from repeated measurements obtained by the same evaluators in similar situations. The higher the consistency, the higher the reliability. Cronbach's alpha was used to assess the reliability of the scale developed in this study. In general, a Cronbach's alpha of >0.8, >0.7, and >0.6 represents very good, good, and acceptable reliability, respectively. However, if the value is below 0.6, the scale needs to be revised (102). As shown in Table 4, the Cronbach's alpha values of the PsyCap scale developed in this study, and its subscales for each dimension, i.e., self-efficacy, resilience, optimism, and hope, are 0.750, 0.631, 0.677, 0.653, and 0.701, respectively, all of which are higher than 0.6. Thus, the scale can be considered reliable.

**Table 4:** Reliability of the PsyCap scale.

**Dimension**	**Cronbach's alpha of subscale**	**Cronbach's alpha of full scale**
Self-efficacy	0.631	0.750
Resilience	0.677	
Optimism	0.653	
Hope	0.701	

#### Validity Test

The four PsyCap dimensions of self-efficacy, resilience, optimism, and hope involve many variables, which are correlated among themselves. These interrelationships not only increase the complexity of analysis, but also result in an overlap in the information reflected by the observation data. Therefore, factor analysis was used to extract common factors from the many variables (103). The Kaiser-Meyer-Olkin (KMO) test of sampling adequacy and Bartlett test of sphericity were used to determine whether the PsyCap scale developed in this study satisfies the requirements of factor analysis. According to Kaiser and Rice (104), if a KMO value >0.8, between 0.7 and 0.8, or between 0.6 and 0.7 is obtained, it would be very suitable, suitable, and barely suitable to conduct factor analysis. If the KMO value is below 0.6, it is not suitable to conduct factor analysis. As shown in Table 5, the KMO value of the PsyCap scale was 0.780, indicating that there are many common factors among variables and it would be suitable to conduct factor analysis. The significance (*p*) of Bartlett test of sphericity is <0.001, indicating that the variables are correlated, which also justifies the use of factor analysis.

**Table 5:** KMO test and Bartlett test of sphericity of the PsyCap Scale.

KMO test		0.780
	Approximate chi-square	2188.322
Bartlett test of sphericity	Degree of freedom	120
	Significance	0.000

The structural validity of a questionnaire indicates the degree of correspondence between a certain structure reflected in the measurement results and the measured values. Factor analysis is an ideal method to assess the structural validity of questionnaires (105). According to Comrey and Lee (106), a questionnaire must meet all three following criteria to have acceptable structural validity: (a) the factor loadings of each item in its own factor should be at least 0.450, (b) those in other factors should be <0.320, and (c) there are at least three items for each factor. Because the PsyCap scale has four dimensions, principal component analysis with four factors and varimax rotation was performed. As can be seen from Table 6, the four common factors explain 51.674% of the variance, the four factors with 16 items have acceptable structural validity, and each item can represent the corresponding factor.

**Table 6:** Rotated factor loading matrix.

**Item**	**Mean**	**Standard deviation**	**Factor**			
			**1**	**2**	**3**	**4**
1. You are confident in the safety of the food you buy.	3.49	0.772	0.753			
2. You are confident to solve food safety problems you may encounter.	3.34	0.840	0.788			
3. You cannot obtain accurate food safety information.	3.47	0.976	0.595			
4. You believe that you can contribute to joint solving of food safety problems.	3.57	0.843	0.586			
5. You learn from any food safety accidents you have experienced, if any, and improve your food safety awareness afterwards.	3.09	1.006		0.725		
6. You seek a solution calmly when encountering food problems.	3.60	0.861		0.737		
7. You take great care to avoid buying defective food products.	3.88	0.991		0.648		
8. You have to a zero-tolerance attitude toward foods with safety risks.	3.64	0.920		0.678		
9. You believe that citizens can play a role in food safety risk management.	3.43	0.916			0.622	
10. You believe that the quality of the vast majority of food in the market is guaranteed.	3.75	0.824			0.697	
11. You believe that food safety is better now than in the past.	3.54	0.888			0.734	
12. You believe that food safety will be better in the future.	3.66	0.860			0.649	
13. You believe that it is meaningful to participate in food safety social co-governance.	3.98	0.837				0.748
14. You believe that food safety will be better in the future despite the persistent occurrence of food safety incidents at present.	3.72	0.814				0.659
15. You will actively learn food safety knowledge to protect your dietary health.	3.86	0.826				0.758
16. You believe that there will be better solutions to food safety issues in the future.	3.68	0.897				0.623

## Results

### Empirical Results for the Values of PsyCap and Its Four Dimensions

The PsyCapvalues of citizens who participate in co-governance and its four dimensions were calculated based on the 16 items measuring the four dimensions of PsyCap given in Table 1. As shown in Table 7, the mean values of the four dimensions are in a favorable range. Accordingly, following Luthans et al. (96), it can be concluded that the respondents had relatively high levels of self-efficacy, resilience, optimism, and hope, and thus high overall PsyCap.

**Table 7:** Empirical results for PsyCap and its four dimensions.

**PsyCap and its four dimensions**	**Min**	**Max**	**Mean**	**Standard deviation**
PsyCap	1.81	4.75	3.6061	0.40466
Self-efficacy	1.25	5.00	3.4684	0.59297
Resilience	1.25	5.00	3.5519	0.67431
Optimism	1.25	5.00	3.5947	0.61122
Hope	1.75	5.00	3.8095	0.61308

### Empirical Results on the Correlation Between the Characteristics and PsyCap of Citizens

The one-way ANOVA results for PsyCap are shown in Table 8, Supplementary Table 1. Significant *F*-values were observed for education (*F* = 2.942, *p* = 0.020), annual income (*F* = 3.452, *p* = 0.008), and health status (*F* = 4.848, *p* = 0.008). Moreover, the homogeneity of variance assumption was met for annual income (*p* < 0.05), but not for education or health status (*p* > 0.05). Therefore, the Tamhane's T2 test was performed for annual income and the LSD test for education and health status for *post-hoc* multiple comparisons. As shown in Table 9, citizens with high school, bachelor's degree, master's degree, or higher education had significantly higher PsyCap to participate in food safety co-governance than those with junior high school or lower education. Citizens with an annual income of 50,000–80,000 yuan or more than 100,000 yuan had significantly higher PsyCap than those with an annual income of 36,000 yuan or less. Citizens in good health had significantly higher PsyCap than those in fair health. Further analysis can be performed using the four dimensions of PsyCap.

**Table 8:** Results for one-way analysis of variance about individual characteristics of citizens who will actively participate in food safety social co-governance.

**Variables**	**Analysis of variance**			
	**Levene statistic**	**Significance**	***F*-value**	**Significance**
PsyCap				
Education	1.881	0.112	2.942	0.020^*^
Personal annual income	3.213	0.013	3.452	0.008^**^
Health status	0.250	0.779	4.848	0.008^**^
Self-efficacy				
Personal annual income	4.512	0.001	5.287	0.000^***^
Health status	1.775	0.170	3.140	0.044^*^
Resilience				
Health status	0.674	0.510	6.623	0.001^**^
Optimism				
Education	0.569	0.685	5.240	0.000^***^
Hope				
Education	4.802	0.001	5.370	0.000^***^
Personal annual income	3.245	0.012	4.197	0.002^**^

*, **, and *** *indicate p < 0.05, p < 0.01, and p < 0.001, respectively*.

**Table 9:** *Post-hoc* multiple comparisons.

**Variable**	***I***	***J***	**MD (*I-J)***	***P***
PsyCap	Junior high school or lower	High school Bachelor's degree Master's degree or higher	−0.13882 −0.17453 −0.13370	0.032^*^ 0.002^**^ 0.049^*^
	36,000 yuan or less	50,000–80,000 yuan More than 100,000 yuan	−0.13829 −0.13523	0.022^*^ 0.013^*^
	Good health	Fair health	0.15056	0.003^**^
Self-efficacy	36,000 yuan or less	50,000–80,000 yuan	−0.26006	0.001^**^
		80,000–100,000 yuan	−0.20232	0.016^*^
	36,000–50,000 yuan	50,000–80,000 yuan	−0.25277	0.013^*^
	Good health	Fair health	0.17870	0.017^*^
Resilience	Good health	Fair health	0.29562	0.001^**^
Optimism	Bachelor's degree	Junior high school or lower	0.28415	0.001^**^
		High school	0.16804	0.012^*^
		Junior college	0.18296	0.001^**^
	Master's degree or higher	Junior high school or lower	0.27897	0.006^**^
Hope	More than 100,000 yuan	36,000 yuan or less	0.24082	0.002^**^
		36,000–50,000 yuan	0.22346	0.033^*^
	Junior high school or lower	High school	−0.26329	0.030^*^
		Junior college	−0.25174	0.032^*^
		Bachelor's degree	−0.37760	0.000^***^

*, **, and *** *indicate p < 0.05, p < 0.01, and p < 0.001, respectively*.

#### Citizen Characteristics and Self-Efficacy

As shown in Supplementary Table 1, results of the Student's *t*-test indicated significantly higher self-efficacy (*t* = −2.386, *p* = 0.017) for females than for males, indicating that females had higher self-efficacy for participating in co-governance. A significant *F*-value was obtained for annual income (*F* = 5.287, *p* = 0.000) in the one-way ANOVA for self-efficacy. However, as shown in Table 2, annual income includes five different levels and met the homogeneity of variance assumption (*p* < 0.05). Therefore, the Tamhane's T2 test was performed for *post-hoc* multiple comparisons to determine the association between different levels of annual income and self-efficacy. As shown in Table 9, significantly higher self-efficacy was observed in citizens with an annual income of 50,000–80,000 yuan than in those with an income of 36,000–50,000 yuan, and also in those with an income of 50,000–100,000 yuan than in those with an income of 36,000 yuan or less. A significant *F*-value was also obtained for health status (*F* = 3.140, *p* = 0.044). Health status includes three different levels and did not meet the homogeneity of variance assumption (*p* = 0.170). Therefore, the LSD test was performed for *post-hoc* multiple comparisons. As shown in Table 8, citizens in good health had significantly higher self-efficacy than those in fair health.

#### Citizen Characteristics and Resilience

As shown in Table 8, a significant *F*-value was obtained for health status (*F* = 6.623, *p* = 0.001) in the one-way ANOVA for resilience. However, the homogeneity of variance assumption was not met (*p* = 0.510). Therefore, the LSD test was performed for *post-hoc* multiple comparisons. As shown in Table 9, citizens in good health had significantly higher resilience than those in fair health.

#### Citizen Characteristics and Optimism

As shown in Table 8, a significant *F*-value was obtained for education (*F* = 5.240, *p* = 0.000) in the one-way ANOVA for optimism. However, optimism includes five different levels and did not meet the homogeneity of variance assumption (*p* = 0.685). Therefore, the LSD test was performed for *post-hoc* multiple comparisons. As shown in Table 9, significantly higher optimism was observed in citizens with bachelor's degree than in those with lower educational attainment, and also in those with master's degree or higher than in those with junior high school or lower education.

#### Citizen Characteristics and Hope

As shown in Table 8, significant *F*-value were obtained for education (*F* = 5.240, *p* = 0.000) and annual income (*F* = 4.197, *p* = 0.002) in the one-way ANOVA for hope. Moreover, the homogeneity of variance assumption was met for both variables (*p* > 0.05). Therefore, the Tamhane's T2 test was performed for *post-hoc* multiple comparisons for both variables. As shown in Table 9, significantly higher hope was observed in citizens with high school, junior college, or a bachelor's degree than in those with junior high school or lower education, and also in those with an annual income of more than 100,000 yuan than in those with an annual income of 50,000 yuan or less.

## Conclusions and Prospects

### Conclusions

This paper has introduced the concept of analyzing PsyCap and its four dimensions and developed a framework for analyzing the relationship between characteristics and PsyCap of citizens willing to participate in food safety social co-governance. Moreover, the 10 most important individual characteristics associating with this behavior, including gender, age, marriage, household size, education, annual income, having children aged under 18 in the household or not, having a pregnant woman in the household or not, health status, and occupation, were identified. On this basis, the PsyCap of citizens with different individual characteristics willing to participate in co-governance was empirically examined based on a survey using Student's *t*-test, one-way ANOVA, and *post-hoc* multiple comparisons. The empirical results show that age, marriage, household size, having children aged under 18 in the household or not, having a pregnant woman in the household or not, and occupation had no significant correlation with any of the four dimensions of PsyCap. Therefore, these individual characteristics are ineffective in influencing the PsyCap of citizens to encourage them to participate in co-governance. The other four individual characteristics, including annual income, education, health status, and gender, are not significantly correlated with all dimensions of PsyCap, and have different associations with PsyCap. The main conclusions are summarized below.

First, gender is only significantly correlated with self-efficacy for participating in co-governance. Specifically, females have higher self-efficacy for participating in food safety co-governance. This is consistent with the findings of Schafer et al. (47) and Haapala and Probart (45). However, gender does not significantly associate with the PsyCap of citizens to encourage participation in co-governance.

Second, annual income is significantly correlated with self-efficacy and hope for participating in co-governance, but it does not significantly associate with resilience or optimism. In this study, significantly higher self-efficacy was observed in citizens with an annual income of 50,000–80,000 yuan than in those with 36,000–50,000 yuan, and also in those with an income of 50,000–100,000 yuan than in those with an income of 36,000 yuan or less. This is similar to the findings of Caprara et al. (74) and Zani and Barrett (75). Significantly higher hope was observed in citizens with an annual income higher than 100,000 yuan than in those with an income of 50,000 yuan or less. This is similar to the findings of Ye (72). Annual income associates with PsyCap through self-efficacy and hope. Moreover, citizens with an annual income of 50,000–80,000 yuan and more than 100,000 yuan had higher PsyCap to participate in co-governance than those with an income of 36,000 yuan or less. This is similar to the conclusions of Cole et al. (34) and Xu et al. (79).

Third, education is significantly correlated with optimism and hope for participating in co-governance, but it does not significantly associate with self-efficacy or resilience. In this study, significantly higher optimism was observed in citizens with a bachelor's degree than in those with lower educational attainment, and also in those with a master's degree or higher than in those with junior high school or lower education. This is similar to the conclusion of Jonge et al. (67). Significantly higher hope was observed in citizens with high school, junior college, or a bachelor's degree than in those with junior high school or lower education. This is similar to the findings of Liu et al. (69) and Babalola (33). Moreover, citizens with high school, bachelor's degree, and master's degree or higher education had significantly higher PsyCap than those with junior high school or lower education. This is similar to the conclusion of Xu et al. (79).

Lastly, health status is significantly correlated with self-efficacy and resilience for participating in co-governance, but it does not significantly associate with optimism or hope. In this study, citizens in good health had significantly higher self-efficacy and resilience than those in fair health. This is similar to the findings of Gase et al. (49) and Lutz et al. (54). Moreover, citizens in good health had higher PsyCap to participate in co-governance than those in fair health. This is similar to the findings of Xu et al. (79).

Previous studies have confirmed that many individual, family, and social characteristics associate with one or more dimensions of citizens' PsyCap as well as overall PsyCap. However, based on a survey in Wuxi, Jiangsu Province, this study reveals that citizens are likely to actively participate in food safety social co-governance only when they have at least one of three characteristics: (a) higher than average income in their city of residence; (b) a bachelor's degree or higher education; or (c) good health. Therefore, motivating citizens to participate in co-governance is a long-term process in China. The fundamental strategy is to (1) develop the economy and increase citizens' incomes, especially low-income groups (for example, increasing the minimum subsistence allowance for low-income groups and especially increasing central and provincial fiscal transfer payments to relatively poor rural areasto increase incomes in these areas); (2) promote education, especially higher education, to improve food safety literacy of the public (for example, by improving compulsory education in relatively poor rural areas and providing as many affordable and practical education and training opportunities as possible to low-income workers); and (3) implement more progressive health policies to improve sanitation and public health. In the short term, it may be effective to strive to increase the income of low-income groups, implement popular education about food safety knowledge, and promote healthy dietary habits and lifestyles.

### Prospects

This study does have some limitations. Due to the lack of a validated scale for measuring the PsyCap of citizens to participate in food safety social co-governance, the reliability, and validity of the scale developed in this study requires further investigation. In addition, the sample for this study was limited to citizens in Wuxi, and the demographics of the sample do not perfectly match the overall demographics of Wuxi^1^. Therefore, further research is required to confirm the findings this study. It is hoped that the methods, conclusions, and policy recommendations of this case study on Wuxi can be promoted across China and provide guidance at the national level. More broadly, it is hoped that the experience of China may provide lessons and insights for other developing countries.

## Data Availability Statement

The original contributions presented in the study are included in the article/Supplementary Material, further inquiries can be directed to the corresponding authors.

## Ethics Statement

Ethical approval for this study and written informed consent from the participants of the study were not required in accordance with local legislation and national guidelines. All data were de-identified and the article does not contain any individual person's data. Therefore, this study was exempted from ethics approval.

## Author Contributions

LW conceptualized the study. KQ processed and analyzed the relevant data, as well as wrote the draft manuscript. XC collected and analyzed the relevant data, wrote, and revised the manuscript. All authors contributed to the study design, interpretation the results and manuscript revision, and have approved the final manuscript.

## Funding

This study was supported by the National Social Science Fund of China: Research on social co-governance of food safety risks and cross-border cooperative governance mechanism (20&ZD117).

## Conflict of Interest

The authors declare that the research was conducted in the absence of any commercial or financial relationships that could be construed as a potential conflict of interest.

## Publisher's Note

All claims expressed in this article are solely those of the authors and do not necessarily represent those of their affiliated organizations, or those of the publisher, the editors and the reviewers. Any product that may be evaluated in this article, or claim that may be made by its manufacturer, is not guaranteed or endorsed by the publisher.

## Footnotes

^1^The demographics of the sample for this study differed from those of the urban population of Wuxi, and, of course, could not represent the overall demographics of China. Therefore, readers should assess the results of this study based on consideration of data representativeness.
