# Supplementary material for: Citizen Characteristics and Their Participation in Food Safety Social Co-governance: Public Health Implications
Source: Front Public Health. 2021 Nov 24;9:772117. doi: 10.3389/fpubh.2021.772117 (PMC8652086; doi:10.3389/fpubh.2021.772117)
Supplement: Supplementary file 1 [file Table_1.docx]

**Supplementary Table 1 |** Results for Student’s t-test about individual characteristics of citizens who will actively participate in food safety social co-governance

| Variables | Student’s t-test | | | |
| --- | --- | --- | --- | --- |
|  | Mean difference | Standard deviation | t-value | Significance |
| **Self-efficacy** | | | | |
| Gender | -.104 | .043 | -2.386 | .017* |

**Note: *, **, and *** indicate p < 0.05, p <0.01, and p < 0.001, respectively.**
